# Dysregulation of arginine methylation in tumorigenesis

**DOI:** 10.3389/fmolb.2024.1420365

**Published:** 2024-06-07

**Authors:** Xiao Li, Yaqiong Song, Weiwei Mu, Xiaoli Hou, Te Ba, Shaoping Ji

**Affiliations:** ^1^ Department of Basic Medicine, Zhengzhou Shuqing Medical College, Zhengzhou, Henan, China; ^2^ Department of Shanxi University of Chinese Medicine, Jinzhong, Shanxi, China; ^3^ Department of Biochemistry and Molecular Biology, Medical School, Henan University, Kaifeng, Henan, China

**Keywords:** histone, arginine methylation, arginine methyltransferases, dysregulation, cancer

## Abstract

Protein methylation, similar to DNA methylation, primarily involves post-translational modification (PTM) targeting residues of nitrogen-containing side-chains and other residues. Protein arginine methylation, occurred on arginine residue, is mainly mediated by protein arginine methyltransferases (PRMTs), which are ubiquitously present in a multitude of organisms and are intricately involved in the regulation of numerous biological processes. Specifically, PRMTs are pivotal in the process of gene transcription regulation, and protein function modulation. Abnormal arginine methylation, particularly in histones, can induce dysregulation of gene expression, thereby leading to the development of cancer. The recent advancements in modification mediated by PRMTs and cancer research have had a profound impact on our understanding of the abnormal modification involved in carcinogenesis and progression. This review will provide a defined overview of these recent progression, with the aim of augmenting our knowledge on the role of PRMTs in progression and their potential application in cancer therapy.

## 1 Introduction

Cancer, also known as malignant tumor, is caused by malignant cell proliferation and is aggressive and metastatic (Valastyan and Weinberg, 2011). Arginine is a positively charged amino acid, and as early as 1967, Paik’s team demonstrated the presence of methyl groups in arginine. Arginine methylation is a PTM with a more stable structure than other epigenetic regulators (Bedford and Clarke, 2009). Protein methylation majorly includes lysine methylation and arginine methylation. Both modifications play important role in cellular processes. Arginine methylation, a crucial PTM of histones and various non-histone proteins, plays a crucial role in a wide range of biological activities, including the regulation of gene expression, signal transduction, cell development, and carcinogenesis (Gao et al., 2024). Histones are modified post-translationally through the action of histone modifying enzymes. One of these modifications is methylation, which is carried out by lysine methyltransferases and/or arginine methyltransferases. These enzymes specifically methylate lysine and arginine residues on histones, respectively. Histones H2A and H2B have been less studied than histones H3 and H4 (Zhang et al., 2003), despite being important components of chromatin structure and regulation.

PRMTs play an increasingly important role in physiological processes and development of diseases (including cancer) by mediating the methylation of arginine residues in histone and non-histone proteins (Shishkova et al., 2017) As researchers discovered the link between protein arginine methylation and disease in the early 2000s, they began to extensively explore the possibility of PRMTs as therapeutic targets (Kim D. et al., 2014).

Within the broad category of mammals, there exists a family of nine separate sequence-related protein arginine methyltransferases, also known as PRMT1-9 (Morales et al., 2016), see Table 1. These PRMTs can be divided into three classes based on their catalytic activity: Type I, which includes PRMT1, PRMT2, PRMT3, CARM1 (Coactivator-associated arginine methyltransferase 1, also known as PRMT4), PRMT6, and PRMT8; Type II, encompassing PRMT5 and PRMT9; and Type III is solely represented by PRMT7 (Bedford, 2007). Type I PRMTs have the capacity to methylate 3-methylhistidine-5-methylarginine (MMA) and asymmetric dimethylarginine (ADMA). PRMT1 is capable of methylating H4R3 (Shia et al., 2012) and H2AR11 (Waldmann et al., 2011) *in vitro*, and PRMT2 can methylate histones H4 (Rowley et al., 2023) and/or H3R8 (Li et al., 2023). PRMT4 methylates H3R2 (Mann et al., 2013), R17 (Hu et al., 2020), and R26 (Mann et al., 2013). PRMT6 has the capacity to methylate not only H4 (Hyllus et al., 2007) and H2AR29 (Waldmann et al., 2011) *in vitro*, but also H3R2 (Wang J. et al., 2023) *in vivo*. Type II PRMTs are responsible for the methylation of MMA and symmetric dimethylarginine (SDMA). PRMT5 methylates H4R3 (Sipos et al., 2017), H2AR3 (Yang et al., 2021), H3R2 (Chen et al., 2017) and H3R8 (Yang et al., 2021). Lastly, Type III PRMTs are unique in that they can only catalyze MMA production. PRMT7 catalyzes the mono-methylation of histones, but the specific target of arginine has not been identified (Lee et al., 2005).

**TABLE 1:** Classification of PRMTs by substrate, sub-cellular location and their functions.

Types	PRMT enzyme	Histone substrate	Cellular location	Arginine methylation	Function
I	PRMT1	H2AR11 (Waldmann et al., 2011) H4R3 (Shia et al., 2012)	Cytoplasm and nucleus	MMA ADMA	Transcriptional regulation (Waldmann et al., 2011; Chen et al., 2017; Yang et al., 2021), signal transduction (Li et al., 2023), RNA splicing (Tarighat et al., 2016), DNA repair (Karkhanis et al., 2012; Strahan et al., 2017), ribosome homeostasis (Chen et al., 2021; Li et al., 2021), cell proliferation and differentiation (Shia et al., 2012; Wang et al., 2023a)
	PRMT2	H3R8 (Li et al., 2023) H4 (Rowley et al., 2023)	Nucleus		
	PRMT3	—	Cytoplasm		
	PRMT4 (CARM1)	H3R2 (Mann et al., 2013) H3R17 (Hu et al., 2020) H3R26 (Mann et al., 2013)	Nucleus		
	PRMT6	H2AR29 (Waldmann et al., 2011) H3R2 (Wang et al., 2023a) H4 (Hyllus et al., 2007)	Nucleus		
	PRMT8	H2A (Lee et al., 2015) H4 (Sayegh et al., 2007)	Plasma membrane		
II	PRMT5	H2AR3 (Sipos et al., 2017) H3R8 (Yang et al., 2021) H3R2 (Chen et al., 2017) H4R3 (Yang et al., 2021)	Cytoplasm and nucleus	MMA SDMA	
	PRMT9	—	Cytoplasm and nucleus		
III	PRMT7	H2AR3 (Karkhanis et al., 2012) H3R2 (Morita et al., 2021) H4R3 (Ying et al., 2015)	Cytoplasm	MMA	
Not classified	PRMT10	—	—	—	
	PRMT11	—	Cytoplasm	—	

Recently, an expansion in the PRMT family has been uncovered by researchers, revealing the presence of two new members, PRMT10 and PRMT11 (Xu et al., 2014; Harada et al., 2015). In this review, we will explore the emerging field of arginine methylation and its associations with tumorigenesis, providing an in-depth understanding of the functional significance and potential molecular pathways of this modification in tumors. The primary objective is to provide the research community with a comprehensive overview of the latest advancements in this specific field of research.

## 2 PRMT mutations involved in cancer

The presence of PRMTs variants could influence the progression of cancer cell phenotypes through their arginine methyltransferase activity. The transcript of *PRMT1* gene undergoes mRNA alternative splicing to generate three splicing variants, collectively referred to as v.1, v.2, and v.3 (Scott et al., 1998). PRMT1 v.1 and v.2 expression is significantly reduced in breast cancer. Notably, PRMT1 v.2 is also demonstrated to augment the survival and invasive properties of breast cancer cells (Adamopoulos et al., 2019). Three novel PRMT2 splicing variants, *PRMT2α*, *PRmt2β*, and *PRMT2γ*, were isolated in breast cancer cells (Zhong et al., 2017). These variants lack distinct motifs and therefore produce distinct C-terminal domains, which directly affect the subcellular localization of PRMT2. Furthermore, the PRMT2 variant is able to bind estrogen receptor α both *in vivo* and *in vitro*, thereby playing a role in the progression of breast cancer (Zhong et al., 2012).

PRMT8 v. 1 and v. 2 were identified in glioblastoma cell (GBM) lines and three breast cell lines. The PRMT8 v.2 exhibited a pronounced ability to localize to the nucleus in U87MG glioblastoma cells (Hernandez and Dominko, 2016). PRMT8 v.1 was observed to be expressed in both the U-2 OS human osteosarcoma and CRL-2073 human teratoid cancer cell lines (Hernandez et al., 2017). Moreover, PRMT9 genetic mutation has been identified as a significant risk factor for familial lung adenocarcinoma (Zhang et al., 2023). A recent study has highlighted the increased activity of PRMT9 in the cells and leukemia stem cells of individuals diagnosed with acute myeloid leukemia (Dong et al., 2024).

## 3 Abnormal arginine methylation in histones

**FIGURE 1:**
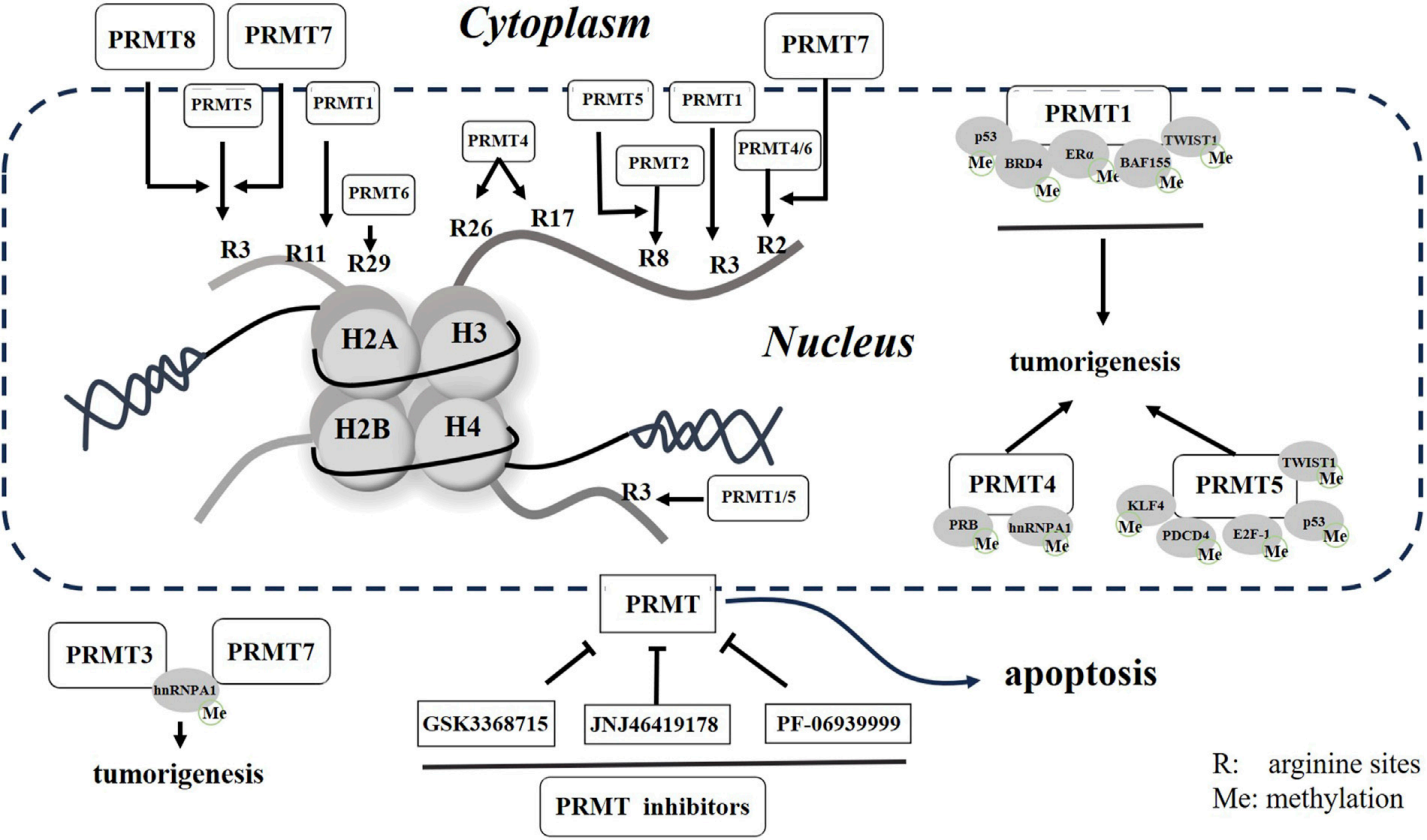
Schematic representation of PRMTs and small molecular inhibitors in cells. Potential arginine residues methylated by PRMTs are presented with numbered R in histones. Both methylated histones to non-histone proteins will promote tumorigenesis, but these processes can be reversed by small molecular inhibitors of PRMTs in cancer.

The modification of histone arginine methylation is a sophisticated PTM that has a significant impact on various cellular processes. Specifically, the histone modifications of H4R3me2a and H3R2me2s are associated with transcriptional activation, whereas the modifications of H4R3me2s, H3R2me2a, and H3R8me2s exhibit inhibitory effects on transcription (Di Lorenzo and Bedford, 2011) (Figure 1).

### 3.1 Aberrant level of arginine methylation in promoter regions

Promoters are key regions in genes that regulate transcription and are affected by a variety of histone modifications. The presence or absence of histone methylation can significantly affect the activation or inhibition of transcription of related genes. PRMT is recruited to the promoter region of the target gene as a costimulator to promote gene expression, and if arginine methylation is absent, it is associated with gene silencing (Wang et al., 2019).

Methylation of histone H4R3 by PRMT1 is associated with transcriptional activation. The splicing isomer of fusion genes in acute myeloid leukemia, known as AE9a, is capable of recruiting the PRMT1 complex to the promoter region of the AE9a-activated gene. This interaction leads to the methylation of the H4R3 residue, thereby promoting the progression of leukemia cells (Shia et al., 2012). PRMT7-mediated histone arginine methylation is a key regulatory mechanism in the development of B-cell lymphoma, which is able to recruit H4R3me1 and H4R3me2 to the B-cell lymphoma 6 promoter and subsequently modify the H4R3 site on the promoter by methylation (Ying et al., 2015).

PRMT2 enhances the Wnt signaling pathway by asymmetric dimethylation of WNT5a promoter H3R8, which in turn stimulates the growth of renal cancer cells (Li et al., 2023). Liu et al. found that knockdown of PRMT5 in colorectal cancer cells can reduce the accumulation of H4R3me2s and H3R8me2s markers, and reduce the CpG methylation level of cyclin dependent kinase inhibitor 2B promoter (Yang et al., 2021).

### 3.2 Other abnormal methylation in genome related to transcription

PRMT1 can induce the asymmetric dimethylation of histone H4R3, which subsequently promotes the transcription of downstream genes. This process contributes to the maintenance of the tumorigenic properties of esophageal squamous cell carcinoma (Zhao et al., 2019). PRMT5 interacts with specific protein 1 in the transcriptional inhibition complex and preferentially silences miR-29b through dimethylation of H4R3, thereby reducing its anti-leukemia activity (Tarighat et al., 2016). The N-terminal of plant homologous domain PHD finger protein 1 has the ability to recognize PRMT7-mediated H4R3 symmetric dimethylation catalyzed by WD repeat domain 77, and this specific recognition promotes tumorigenesis (Liu et al., 2018).

The expression of PRMT4 induced by high glucose increased the apoptosis of retinal pigment epithelial cells through asymmetric dimethylation of H3R17 (Kim D. I. et al., 2014). Mann et al. found that the interaction of Aminophile, glutamate and leucine-rich protein 1 and PRMT4 promotes the histone H3 arginine dimethylation, which in turn regulates epigenetic changes during breast cancer progression (Mann et al., 2013).

PRMT9 specifically recognizes and targets the R41 and R43 proteins as catalysts for arginine methylation, thereby reducing the activity of the mitochondrial antiviral signaling protein gene (Bai et al., 2022). In Hepatocellular carcinoma (HCC), PRMT9 increases its arginine methylation at R76 and R100 by targeting member 8 of the human heat shock homologous protein family A, accelerates the progression of HCC *in vivo* (Deng et al., 2023).

The expression level of PRMT5 in hepatocellular carcinoma is significantly high, and an increase in its overexpression can lead to an elevation in the level of H2A/H4 symmetric dimethylation (Sipos et al., 2017). H2AR29 is specifically enriched in PRMT6-inhibited genes, suggesting that H2AR29me2 may be involved in transcriptional repression (Waldmann et al., 2011). In GBM, PRMT6 promotes transcription through histone methylation marker H3R2me2a, which in turn promotes the development of GBM (Wang J. et al., 2023). PRMT7 specifically methylates histone H2AR3 (Karkhanis et al., 2012) and H3R2 (Morita et al., 2021), thus playing a role in regulating cellular responses to DNA damage and the global transcription process of genome activation.

## 4 Dysregulation of gene expression involved in arginine methylation in cancer

PRMT3 may also play a role in carcinogenesis by regulating specific gene expression within cancer cells. Other scientists have recently discovered that the PRMT3 protein plays a vital role in chemotherapy resistance in cancer. Shi et al. found that PRMT3-mediated insulin-like growth factor 2 mRNA binding protein 1 arginine methylation at the R452 site promoted oxaliplatin resistance in HCC (Shi et al., 2023). Wang et al. also confirmed that PRMT3 interacts with methyltransferase 14 and participates in its arginine methylation, promoting malignant progression and treatment resistance of endometrial cancer (Wang Y. et al., 2023). In the study of drug resistance in pancreatic cancer, PRMT3 enhances chemotherapy resistance in pancreatic cancer by methylating the RNA recognition motif of heterogeneous nuclear ribonucleic acid protein 1, thereby increasing the expression of G-member 2 of the ATP-binding cassette subfamily (Hsu et al., 2018).

PRMT4 specifically modifies additional sex combs like 2 at residue 639/641 through methylation, resulting in a diminished tumor inhibitory function of the mixed lineage leukemia protein 3 (Zhao et al., 2022). Several independent research efforts have uncovered that PRMT5 plays a pivotal role in the regulation of genes that either stimulate or suppress tumor growth. At R361, PRMT5 mechanically triggers Deleted in Pancreatic Cancer 4 methylation, promoting colorectal cancer metastasis (Liu A. et al., 2023). In breast, colorectal, and prostate cancer cells, PRMT4, PRMT5, and PRMT7, as well as their mediated heterogeneous nuclear ribonucleoprotein A1 methylation and splicing isoforms, can effectively promote the growth of cancer cells (Li et al., 2021).

Liu et al. (2020) were the first to find elevated levels of PRMT7 expression in renal cancer tissues. PRMT7 regulates the expression of the proto-oncogene c-MYC and has been shown to promote renal cancer cell growth and accelerate tumor development in a c-MYC dependent manner. PRMT9 is also referred to as F-box unique protein 11 (FBXO11). In contrast to the previously observed high expression, PRMT9 levels were found to be significantly decreased in osteosarcoma and prostate cancer (Grypari et al., 2023). PRMT11, also known as FBXO10, is less studied now, but previous studies have shown that human breast cancer susceptibility-related SNP rs7042509 is present in PRMT11. Xu et al. further found that cellular stress can upregulate PRMT11, and suggested that lens epithelial-derived growth factors may play an important role in this process (Xu et al., 2014). However, in mice, loss of function of PRMT11 did not have a significant effect on B-cell leukemia-lymphoma 2 or B lymphocyte accumulation (Masle-Farquhar et al., 2021).

## 5 Arginine methylation in signal transducers

PRMT1-mediated methylation and activation of phosphoglycerate dehydrogenase enhanced serine synthesis and ameliorated oxidative stress, thereby stimulating the growth and proliferation of HCC cells both *in vivo* and *in vitro* (Wang K. et al., 2023). As demonstrated by Liu J. et al. (2023), PRMT1 is crucial in directly methylating cGAS and blocking cGAS/STING sensing signals, thereby facilitating tumor immune evasion. PRMT1 also has the ability to suppress P53-induced transcriptional activity through methylation at multiple regions, thus protecting breast cancer cells from undergoing apoptosis or senescence (Liu et al., 2021).

Studies have shown that high expression of PRMT3 is associated with more aggressive brain cancers. PRMT3 can significantly promote the development and progression of GBM by enhancing glycolysis and expression of hypoxia-inducible factor 1α (Liao et al., 2022). In terms of the mechanism of colorectal cancer tumorigenesis, a specific enzyme, hypoxia-inducible factor 1α R282 methylation, is catalyzed by PRMT3. This process reduces the level of polyubiquitination of hypoxia-inducible factor 1α, leading to a potential stabilization of tumorigenesis in colorectal cancer (Zhang et al., 2021). Moreover, PRMT3 may play a critical role in the regulation of C-creatine, which may contribute to the promotion of tumorigenesis (Hu et al., 2021). Analysis showed that the role of PRMT3 in tumorigenesis is highly dependent on the availability of methyl groups. Du et al. performed research that demonstrated PRMT4 promotes the progression of HCC cells by activating the AKT/mTOR signaling pathway (Du et al., 2021).

Jiang et al. also discovered that activation of the AKT/mT0R pathway caused a significant increase in PRMT6 expression in endometrial cancer, thereby exhibiting a carcinogenic effect (Jiang et al., 2020). PRMT6 acts as a regulator of glucose metabolism through PTM, contributing to the progression of lung cancer (Sun et al., 2023). PRMT7 hinders the proliferation and migration of gastric cancer cells by directly affecting the missing phosphatase and tensin homologues(PTEN) on chromosome 10 (Wang X. et al., 2023).

Research has demonstrated that PRMT9 contributes significantly to the progression of liver cancer and lung cancer through upregulation of the PI3K/Akt/GSK-3β/Snail signaling pathway, leading to enhanced invasion and metastasis (Jiang et al., 2018). PRMT10 is highly expressed in the prostate and has a regulatory effect on the growth of prostate cancer cells by controlling androgen receptor signaling (Harada et al., 2015).

## 6 Arginine methylation in other non-histones

Numerous non-histone proteins have been identified as containing methylated arginine, which are affected by PRMTs through modification of arginine residues on these proteins and play a role in cellular development and tumorigenesis.

In ovarian cancer tissues, PRMT1 overexpression may be involved in tumorigenesis by mediating asymmetric methylation of bromine-containing domain protein 4 (Liu Y. et al., 2023). Arginine methylation plays a crucial regulatory role in E2F transcription factor 1(E2F-1) stability and target gene expression. PRMT4-mediated arginine methylation of retinoblastoma protein is responsible for the dissociation of E2F-1, which subsequently stimulates the transcriptional activation of E2F-1 in dividing cells. This specific methylation negatively regulates the tumor suppressive function of retinoblastoma protein and influences the cell cycle (Kim et al., 2015). However, the reduced arginine methylation of E2F-1 by PRMT5 in tumor cells stabilizes E2F-1, subsequently leading to apoptosis and tumorigenesis (Cho et al., 2012). PRMT5 can modify p53 through arginine methylation, altering its nuclear localization and activity, thereby promoting the occurrence of lymphoma. In addition, PRMT5 can also methylate the zinc finger transcription factor arginine to promote the occurrence of breast cancer tumors. As a common substrate of PRMT1 and PRMT5, Twist-related protein 1 arginine methylation plays a key role in the carcinogenesis and metastasis of head and neck squamous cell carcinoma (Fan et al., 2020). Programmed cell death 4 co-expression with PRMT5 accelerates the growth of breast cancer cells (Kong et al., 2023). Arginine methylation of Heterogeneous nuclear ribonucleic acid protein K can reduce the apoptosis of U-2 OS osteosarcoma cells (Chen et al., 2021).

Despite this, the lack of reliable antibodies and clear mechanisms of demethylases and recognition proteins has, to some extent, hindered the full understanding of the molecular mechanisms and biological functions of arginine methylation.

## 7 Some PRMT inhibitors

Due to reports showing that abnormal levels of PRMT are associated with a variety of pathological conditions, researchers are currently working on selective PRMT inhibitors.

Hu et al. (2015) identified a set of cyanine compounds that are effective at suppressing PRMT1, a critical protein involved in cancer development. Through cell proliferation tests, they found that these compounds can significantly inhibit the growth of three leukemia cell lines. Importantly, the PRMT1 inhibitor GSK3368715 has entered phase I clinical trials, and its main tumor types include pancreatic cancer, non-small cell lung cancer, bladder tumors, and blood tumors of diffuse large B-cell lymphoma. In addition, PRMT5 inhibitors JNJ46419178, GSK3326595 and PF-06939999 are also under study. Among them, GSK3326595 has entered clinical phase II research, which has good brain permeability and anti-tumor effects.

## 8 Conclusion and prospect

The modulation of various biological processes for cell maintenance are mediated by most PRMTs methylation substrates, underscoring the potential implications of arginine methylation dysregulation in the genesis and progression of cancer. PRMTs possesses the capacity to regulate gene expression and protein activity, and has been linked to the pathological procession involved in a multitude of human cancers and inflammation in numerous research studies. Epigenetic regulation is an integral mechanism influencing cell function, predominantly through the induction or repression of histone modifications via PRMTs deposition, thereby enhancing or suppressing expression of key oncogenes, respectively. In summary, it was well known that the expression levels of PRMT1-6 are frequently elevated in tumor tissues and their overexpression is often associated with a poor prognosis. In addition, it was also noted that PRMT7-9 is highly expressed in a majority of tumor specimens, although a minor proportion demonstrates low levels of its expression. As such, PRMTs inhibitors have emerged as a critical role in the global research endeavor to identify efficacious cancer treatment strategies.

In summary, extensive scientific evidence supports the role of arginine methylation in critical biological processes such as transcription, mRNA splicing, DNA damage signaling, and immune signaling. Elevated levels of PRMTs and its variants have been identified in a variety of cancer types, and these associations are frequently linked to increased tumorigenesis and poor clinical outcomes.

## Abbreviations

ADMA, asymmetric dimethylarginine; SDMA, symmetric dimethylarginine; MMA, mono methylarginine; PRMTs, protein arginine methyltransferases; PTM, post-translational modification; GBM, glioblastoma cell; HCC, hepatocellular carcinoma; E2F-1, early 2 factor transcription factor 1.
